# Capabilities and Limitations of ChatGPT in Anatomy Education: An Interaction With ChatGPT

**DOI:** 10.7759/cureus.69000

**Published:** 2024-09-09

**Authors:** Sandeep Saluja, Sarika R Tigga

**Affiliations:** 1 Anatomy, Amrita School of Medicine, Faridabad, IND; 2 Anatomy, University College of Medical Sciences & Guru Teg Bahadur Hospital, New Delhi, IND

**Keywords:** anatomy, anatomy education, artificial intelligence, chatbot, chatgpt, medical students

## Abstract

Background: The growing interest in using ChatGPT (OpenAI, San Francisco, CA) in the medical field highlights the need for in-depth knowledge of its potential and constraints, especially when it comes to anatomy education (AE). Because of its sophisticated natural language processing abilities, it can understand the nuances of anatomical concepts, provide advanced as well as contextually relevant information, and could be a helpful tool for medical students and educators. This study aimed to analyze the capabilities and limitations of ChatGPT and its best possible application in AE.

Methodology: The study incorporated 34 questions that were inquired to ChatGPT after acquiring an online subscription to the 4th version. The questions were arbitrarily formulated after consensus among the researchers. The chatbot’s replies were recorded and evaluated with reference to perfection, validity, and appropriateness.

Results: ChatGPT was observed to be a useful interactive tool for medical students to comprehend the clinical importance and characteristics of anatomical structures. The chatbot clarified the anatomical basis of ischemic heart disease and adequately tabulated the differences between the arteries and veins. Even though ChatGPT-4 was able to produce images of different anatomical structures, it fell short of accurately displaying the necessary features. Further, the chatbot generated quizzes, including multiple-choice, true-false, fill-in-the-blank, matching, and case-based questions, formulated a relevant overview of the lecture, and also analyzed answers to anatomy questions with adequate reasoning.

Conclusions: ChatGPT can be a useful educational resource for medical students with the potential to play a crucial role in AE if employed in a methodical way. It imparts significant aid to the anatomy teachers during the execution of the medical curriculum and enhances their jobs while it never takes the place of an educator.

## Introduction

Artificial intelligence (AI) is a large field of computer science associated with making clever machines that have the potential to carry out functions that classically necessitate human intelligence [1]. These functions include the capacity to assimilate, adjust, justify, recognize, and understand various postulations as well as the awareness of intricate human traits such as contemplation, feeling, and imagination [2,3]. ChatGPT (OpenAI, San Francisco, CA), an AI-based large language model (LLM) that was introduced on November 30, 2022, is trained on enormous text datasets in a variety of languages and can produce responses to text input that resemble those of a human [3]. The reinforcement learning from human feedback (RLHF) technique was used in the development of ChatGPT (a generative pre-trained transformer) to train the language model, making it very conversational [4].

ChatGPT's influence on natural language processing (NLP) is immense, and its ongoing development is anticipated to have a major impact on the field of conversational AI in a number of areas [5]. AI, such as ChatGPT, is being used more and more by medical professionals to improve productivity in various domains, including diagnosis, medical imaging interpretation, predictive models, and personalized treatment [6]. ChatGPT has the potential to enhance these applications by integrating conversational skills with medical knowledge. Its capacity to communicate coherent and relevant information highlights its potential as an important tool for medical education and learning aid as well [7]. It offers a number of benefits in improving teaching and learning methodologies, like fostering individualized and interactive learning and formative evaluation procedures. AI has the potential to revolutionize medical education by drastically altering existing methods of teaching and having a significant impact on several domains of medical science [8].

Anatomy has been a cornerstone of medical education for hundreds of years. Anatomical knowledge is crucial for the successful performance of diverse invasive procedures as well as for the diagnosis and treatment of various diseases [9]. The intensive workload of anatomy departments worldwide would be significantly reduced by the use of AI in the anatomical sciences, where cadaveric dissection is still a cornerstone of anatomy and medical education [10]. Nevertheless, these AI aids should be employed carefully and scrutinized for the credibility of information.

Numerous research studies have been carried out to evaluate ChatGPT's effectiveness in several medical fields, including medical examinations and clinical treatment [8]. In light of this, we plan to assess the capability of ChatGPT in various domains of human anatomy. The present study submitted a few prompts to ChatGPT-4 to explore its current potential and optimal utilization in anatomy education (AE) for medical students as well as teachers.

## Materials and methods

In the present study, ChatGPT (4th version) was used, which is considered better than earlier versions. A variety of questions were asked to ChatGPT to investigate its capabilities for AE. After the response to our first question regarding the role of ChatGPT in AE, a series of questions were submitted. The questions were framed from various subdivisions of anatomy, such as general anatomy, clinical anatomy, osteology, histology, embryology, genetics, neuroanatomy, and gross anatomy, including different regions of the body. These questions challenged the chatbot to exhibit its capability to describe, enumerate, tabulate, and explain the clinical significance of various anatomical structures, elucidate the anatomical basis of the diseases, illustrate with diagrams, and create quizzes in the form of multiple-choice, true-false, matching and fill in the blank questions. Additional questions were incorporated to scrutinize ChatGPT’s potential role in teaching-learning and assessment methodologies (Table 1). These questions were chosen principally from competencies mentioned in “Competency Based Undergraduate Curriculum for the Indian Medical Graduate 2018,” guided by the National Medical Commission (NMC) after common consent among the investigators. The responses generated by ChatGPT were recorded in their original form without making any alterations. Both researchers conducted the assessment of the responses of ChatGPT regarding their pertinence, precision, and perfection.

**Table 1:** Questions asked to ChatGPT. AI: artificial intelligence; MCQ: multiple-choice question; DOAP: Demonstration-Observation-Assistance-Performance; AETCOM: attitude, ethics, and communication.

Question number	Questions
1	As an AI-based large language model (LLM), describe your role in anatomy education for MBBS students and faculty briefly.
2	Are you aware of the “Competency Based Undergraduate Curriculum for the Indian Medical Graduate” guided by the National Medical Commission (NMC)?
3	Can you tell what is competency number AN1.2 exactly as mentioned in the "Competency Based Undergraduate Curriculum for the Indian Medical Graduate"?
4	Can you write a short note on “Parts and blood supply of a long bone” and draw a suitable schematic diagram also?
5	Could you tabulate the general differences between arteries & veins?
6	Enumerate peculiarities of the clavicle.
7	Describe factors maintaining arches of the foot with its importance.
8	Can you please write a model answer to the five-mark question to be written by 1^st^ MBBS students: “Describe the anatomical basis of ischemic heart disease.”
9	Explain the clinical significance of pterygoid venous plexus.
10	Mention the clinical importance of Calot’s triangle.
11	Draw & label the transverse section of pons at the upper and lower levels.
12	Enumerate parts & major connections of basal ganglia & limbic lobe.
13	Describe the ultrastructure of muscular tissue with a diagram.
14	Describe the Lyon's hypothesis.
15	Describe cleavage and formation of blastocyst.
16	Please make five multiple-choice questions with explanatory answers to assess the MBBS students for competency number AN 10.3 “Describe, identify and demonstrate formation, branches, relations, area of supply of branches, course, and relations of terminal branches of brachial plexus.”
17	Can you formulate five true or false questions with answers to assess the MBBS students for competency number AN 15.3 “Describe and demonstrate boundaries, floor, roof, and contents of the femoral triangle”?
18	Please generate five matching questions with answers to assess the MBBS students for competency number AN 18.4 “Describe and demonstrate the type, articular surfaces, capsule, synovial membrane, ligaments, relations, movements and muscles involved, blood and nerve supply, and bursae around the knee joint.”
19	Make a short answer question with its answer for competency number AN 24.3 “Describe a bronchopulmonary segment.”
20	Can you please create a case-based MCQ with an explanatory answer on “lateral medullary syndrome” to assess the anatomical knowledge of MBBS students? (Competency Number: AN 58.4)
21	Make five questions with answers for structured oral viva voce to assess the anatomical knowledge of MBBS students for competency number AN 63.1 “Describe & demonstrate parts, boundaries & features of IIIrd, IVth, & lateral ventricle.”
22	Please generate five "fill in the blank" questions with answers to assess MBBS students for NMC competency AN 38.1 “Describe the morphology, identify the structure of the wall, nerve supply, blood supply, and actions of intrinsic and extrinsic muscles of the larynx.”
23	Can you please describe the methods to assess the skill of MBBS students for competency number AN 12.7 “Identify & describe course and branches of important blood vessels and nerves in hand”?
24	Could you provide steps to conduct a DOAP session for MBBS students in respect of competency number AN 33.3 “Describe & demonstrate articulating surface, type & movements of temporomandibular joint”?
25	Are you aware of AETCOM for MBBS Professional-1 directed by NMC?
26	Can you suggest steps to conduct an introductory session of two hours for AETCOM module 1.5 competency: “Demonstrate respect and follow the correct procedure when handling cadavers and other biologic tissues”?
27	Can you make a lecture for a PowerPoint presentation for competency number AN 44.3 “Describe the formation of the rectus sheath and its contents”?
28	Are you able to evaluate and give marks to the written answer of an MBBS student for any question?
29	For example, in the question, "Describe arterial supply of uterus", the student's answer is “Uterus is supplied by uterine and ovarian artery". What is your evaluation or feedback for this answer or how many marks out of 5 you will give for this answer?
30	Can you identify the structure in the given picture?
31	Can you identify the structure in the given picture?
32	Can you identify the red arrow-marked part of the stomach in the given picture?
33	Could you please propose some techniques to get accurate, reliable, and valid answers from you to improve anatomy education?
34	Thanks. We hope that our discussion may be useful for the growth of anatomy.

## Results

**Figure 1:**
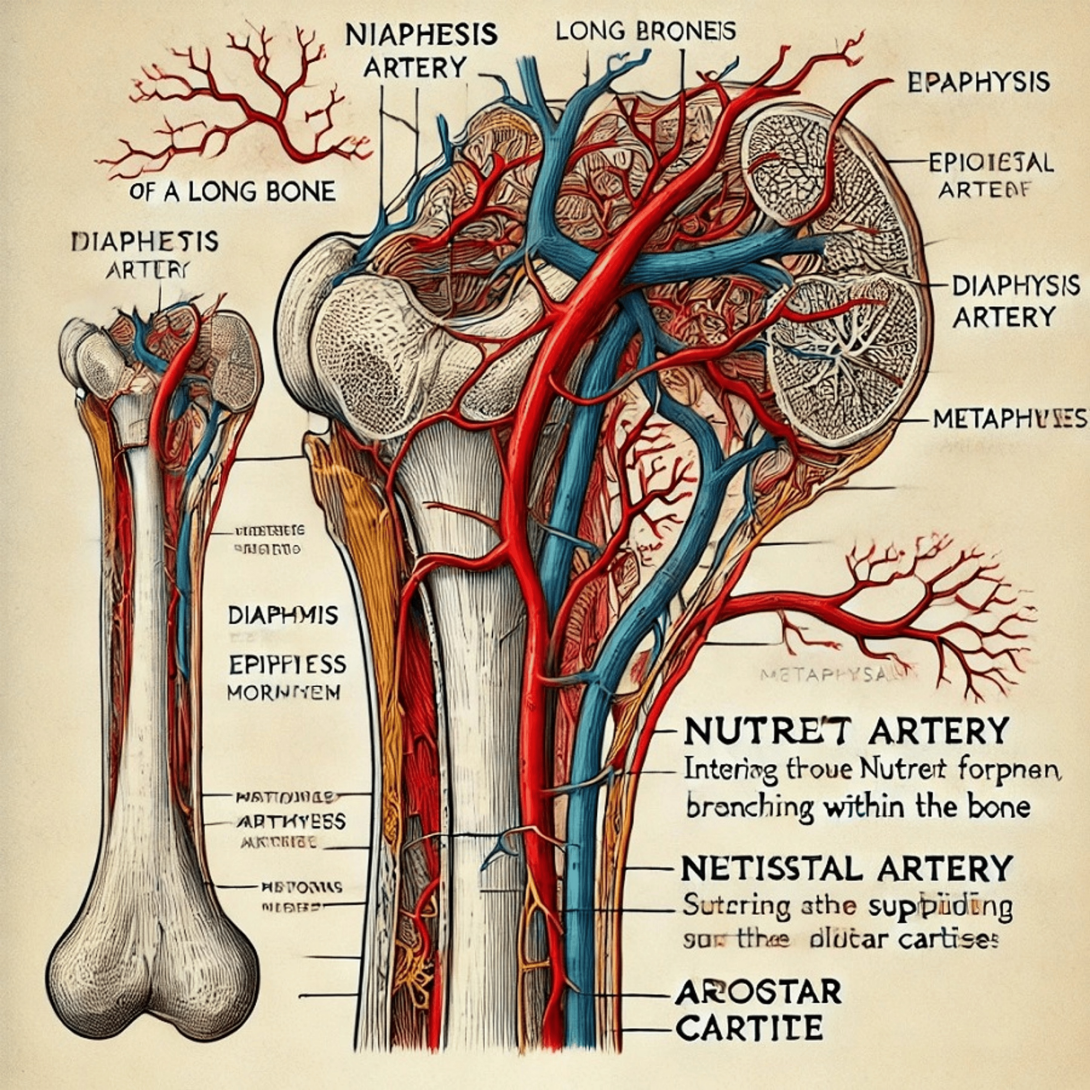
Diagram of parts and blood supply of a long bone created by ChatGPT.

**Figure 2:**
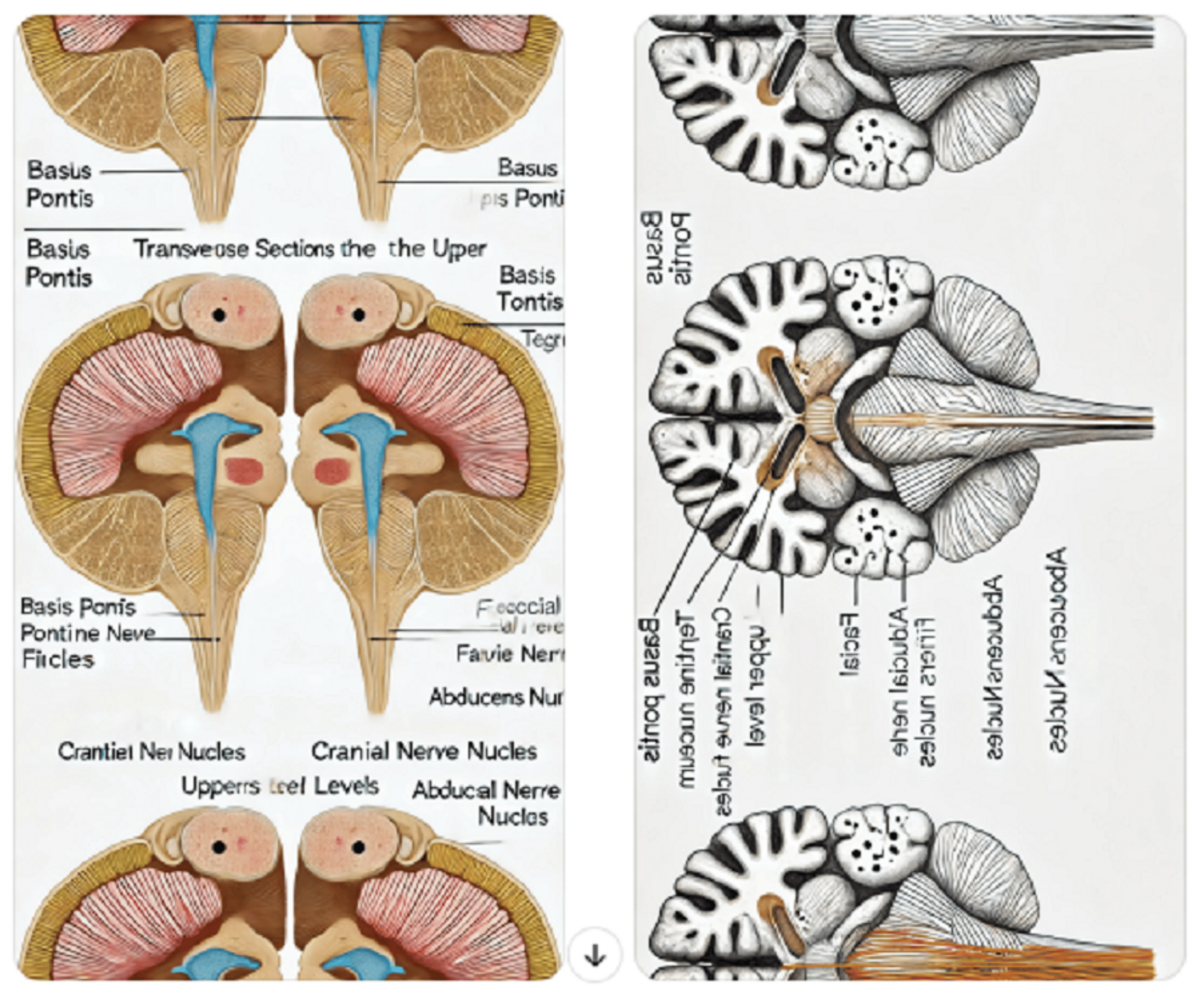
Diagram of transverse section of pons at the upper and lower level generated by ChatGPT.

**Figure 3:**
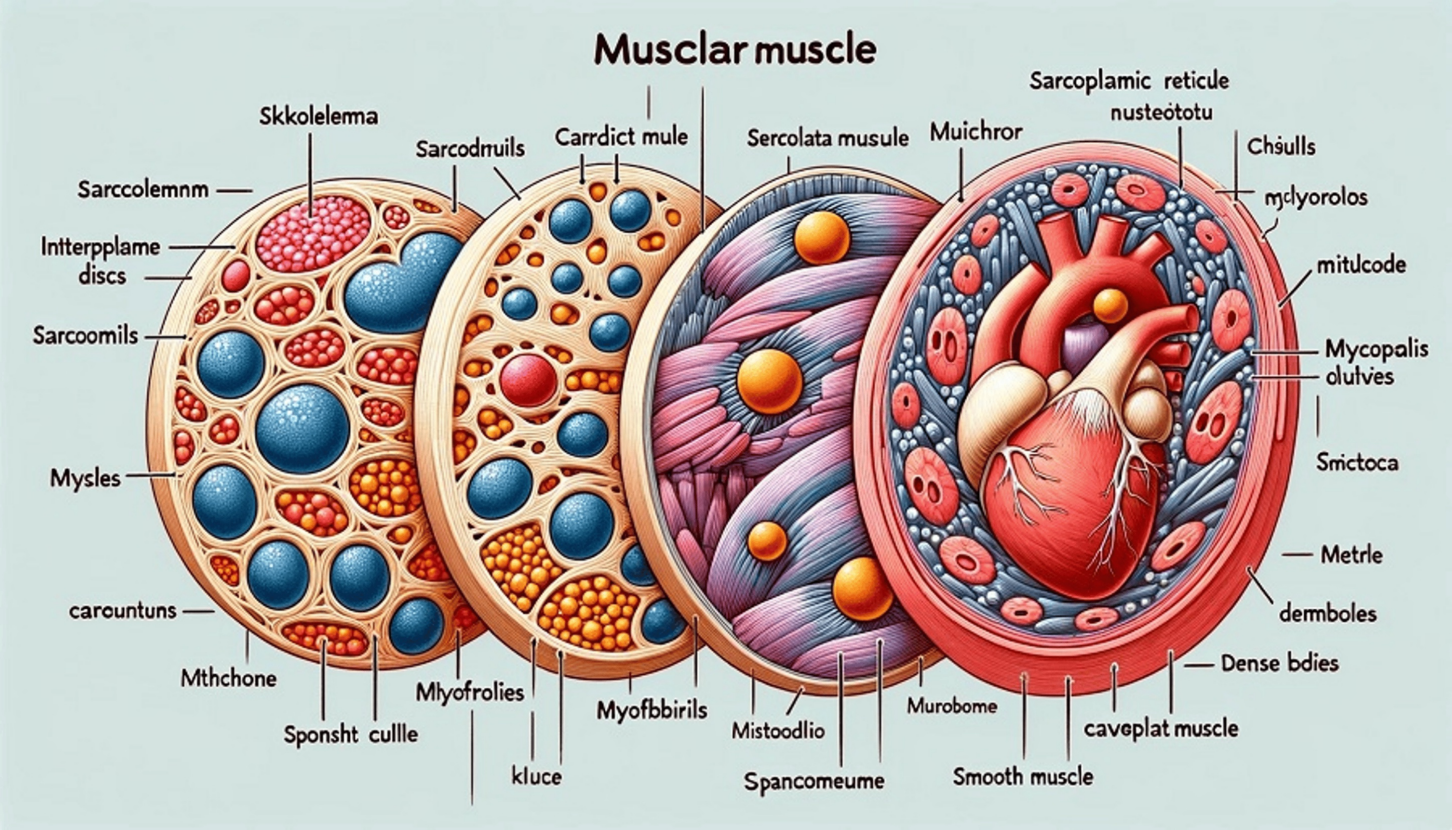
Diagram of ultrastructure of muscular tissue made by ChatGPT.

**Figure 4:**
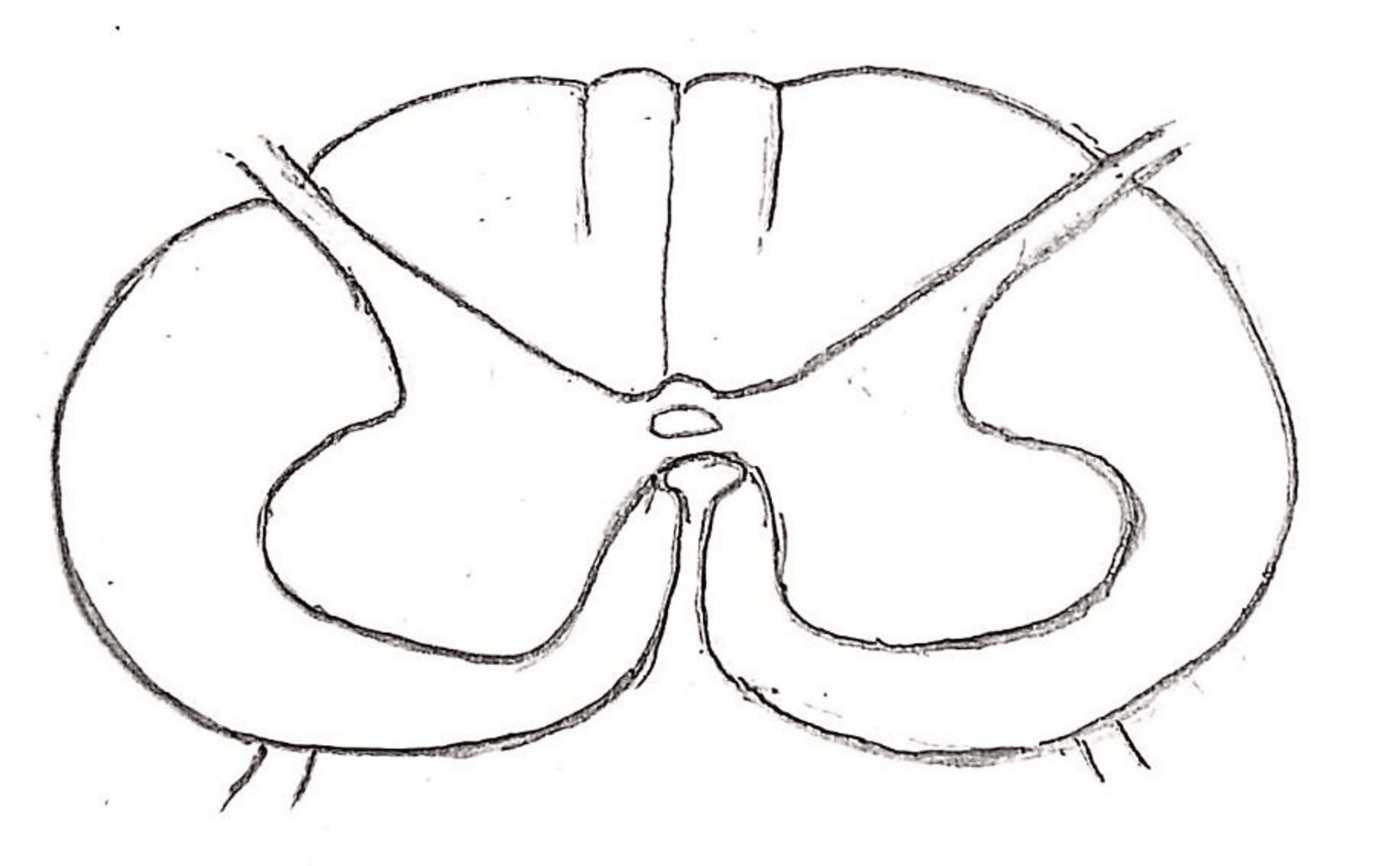
Picture of an outline of the spinal cord submitted to ChatGPT for identification.

**Figure 5:**
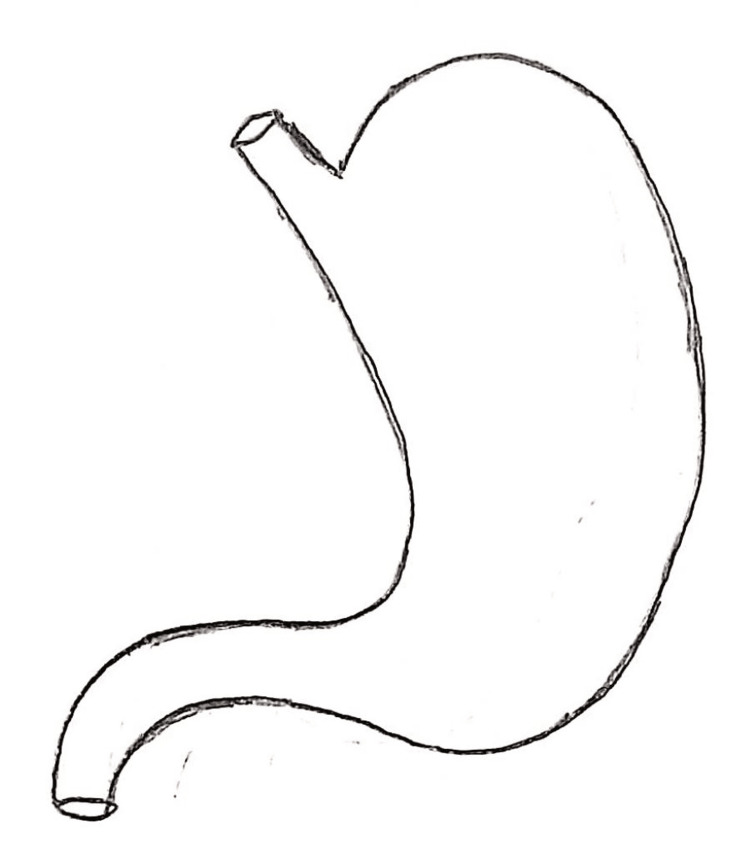
Picture of an outline of the stomach submitted to ChatGPT for identification.

**Figure 6:**
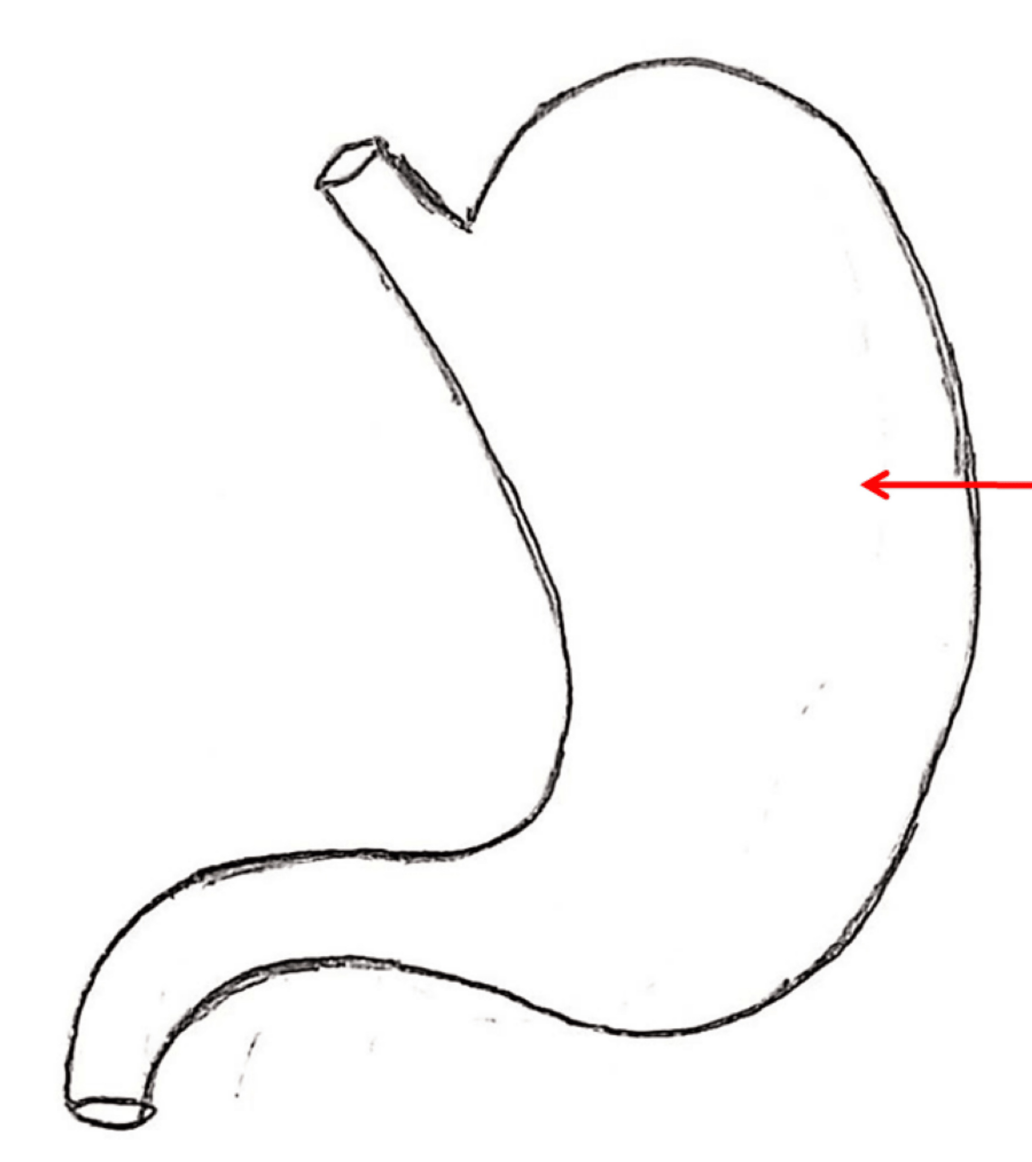
Picture of an arrow-marked part of the stomach submitted to ChatGPT for identification.

A total of 34 questions were asked and the responses produced by ChatGPT-4 were noted without doing any modification. The chatbot was observed to be aware of the "Competency Based Undergraduate Curriculum for the Indian Medical Graduate." It produced the diagrams of the parts and blood supply of a long bone, the transverse section of pons at the upper and lower levels, and the muscular tissue (Figures 1-3). The chatbot also created a wide range of quizzes, including multiple-choice, true-false, fill in the blank, matching, and case-based questions, formed an outline of the lecture, and analyzed the answer to the anatomy question. Furthermore, ChatGPT was asked to identify the hand-drawn schematic diagram of the transverse section of the spinal cord, the outline of the stomach, and its arrow-marked central part (Figures 4-6). The majority of the chatbot's responses were clear, precise, and generated in a systematic manner (Table 2).

**Table 2:** Responses generated by ChatGPT.

Question number	Responses
1	As an AI-based large language model, I can serve as a valuable tool in the education of MBBS students and faculty by offering several benefits: Supplementary Learning Resource: I can provide explanations, descriptions, and detailed breakdowns of complex anatomical structures and concepts, enhancing the learning materials already used in courses. Interactive Q&A: I can answer questions on the fly, offering students and faculty immediate clarification on topics they find challenging. This can be especially helpful for reinforcing learning outside of the classroom or during revision periods. Custom Content Creation: I can help create customized study materials like quizzes, summaries, and flashcards tailored to the specific needs of students or the curriculum goals set by faculty. Visualization Aid: Through integration with tools like DALL-E, I can generate anatomical images, diagrams, or flowcharts, which can help in visual learning and teaching, making complex information more accessible and easier to understand. Up-to-Date Information: I can provide the latest research findings or updates in the field of anatomy, ensuring that both students and faculty have access to the most current knowledge base. Accessibility: I'm available 24/7, offering consistent educational support to students and faculty, irrespective of their geographical or time constraints. In all, I act as a supportive, versatile educational tool that complements traditional teaching methods, enhancing both teaching efficiency and learning outcomes in anatomy education for MBBS programs.
2	Yes, I'm aware of the "Competency Based Undergraduate Curriculum for the Indian Medical Graduate," which is guided by the National Medical Commission (NMC) in India. This curriculum is designed to emphasize competency-based training that focuses on the development of specific skills, behaviors, and attitudes necessary for Indian medical graduates. The approach aims to ensure that graduates are not only knowledgeable but also skilled at applying their knowledge in practical, real-world medical and healthcare settings. This curriculum incorporates interactive and dynamic teaching methods, including integrated teaching and assessment of both theoretical knowledge and clinical skills.
3	Searched 2 sites “Competency Based Undergraduate Curriculum for the Indian Medical Graduate AN1.2 competency detail” bing.com National Medical Commission — UG Curriculum | NMC nmc.org.in Competency AN1.2 in the "Competency Based Undergraduate Curriculum for the Indian Medical Graduate" pertains to the understanding of the composition of bone and bone marrow. This competency requires students to learn about the general features of bones and joints as part of their anatomy studies. This information is integral for forming a foundational understanding of human skeletal anatomy​ (National Medical Commission).
4	A long bone is structurally divided into several parts: the diaphysis (shaft), epiphyses (ends), metaphyses (narrow portions between the diaphysis and epiphyses), and the articular cartilage covering the joint surfaces. The diaphysis is mainly composed of compact bone that provides strength, while the epiphyses consist of spongy bone that aids in stress distribution. The blood supply to a long bone is primarily through the nutrient artery, which enters the diaphysis through the nutrient foramen. This artery branches into ascending and descending branches, supplying the inner two-thirds of the cortex and the marrow cavity. The epiphyses and metaphyses are supplied by smaller arteries derived from the periosteum, which provide blood to the outer cortex and marrow space. Additionally, the metaphyses and epiphyseal regions have their own separate vascular supply that is critical during growth. I will now create a schematic diagram to illustrate these parts and their blood supply. Here is the schematic diagram illustrating the parts and blood supply of a long bone. This visual aid shows the diaphysis, epiphyses, metaphyses, articular cartilage, and the various arteries including the nutrient artery and periosteal arteries (Figure 1).
5	Here's a table summarizing the general differences between arteries and veins: Feature Arteries Veins Wall Thickness Thick, muscular walls Thinner walls Lumen Size Smaller lumen relative to vein of same size Larger lumen relative to artery of same size Blood Pressure High pressure (due to direct pumping by heart) Lower pressure Blood Flow Away from the heart Towards the heart Valves No valves (except for semi-lunar valves at heart) Valves present to prevent backflow Oxygen Status Typically carry oxygenated blood (except pulmonary arteries) Typically carry deoxygenated blood (except pulmonary veins) This table helps highlight the structural and functional contrasts between these two types of blood vessels.
6	The clavicle, or collarbone, has several peculiarities that distinguish it from other bones: S-Shaped Curve: The clavicle has a double curve, forming an S-shape, which helps in the transmission of forces from the upper limb to the axial skeleton. Subcutaneous Position: It is one of the few bones that lies just under the skin, making it palpable along its entire length. No Marrow Cavity: Unlike most bones, the clavicle is filled with cancellous bone instead of having a marrow cavity. First Bone to Ossify: It is the first bone to begin the process of ossification during fetal development, but one of the last to complete ossification. Articulations: It articulates medially with the manubrium of the sternum at the sternoclavicular joint and laterally with the acromion of the scapula at the acromioclavicular joint. Fracture Site: Due to its location and structure, it is one of the most frequently fractured bones in the body. Function: It serves as a strut between the shoulder blade and the sternum, allowing for arm mobility while protecting neurovascular structures underneath. These features contribute to its unique structural and functional roles in the human body.
7	The arches of the foot are maintained by several factors: Shape and Arrangement of Bones: The unique shapes and the interlocking of foot bones create and maintain the arches. Ligaments: Strong ligaments connect the bones of the foot and help maintain the arches' integrity. Muscles: Both intrinsic and extrinsic muscles of the foot contribute to arch support by dynamically controlling tension. Plantar Fascia: This thick, fibrous band of tissue extends from the heel to the toes, providing critical support to the longitudinal arch. Importance: Weight Distribution: Arches help in distributing body weight evenly across the feet. Shock Absorption: They act as natural shock absorbers during walking, running, or jumping. Efficient Movement: Arches provide leverage and stability, enhancing the efficiency of foot movements.
8	A model answer for describing the anatomical basis of ischemic heart disease (IHD) for a 1st MBBS student could be structured as follows: Ischemic heart disease is primarily caused by reduced blood flow to the myocardium, usually due to atherosclerosis in the coronary arteries. Atherosclerotic plaques narrow these arteries, diminishing the oxygen and nutrient supply to heart muscle cells. The heart's high metabolic demand makes it particularly vulnerable to reductions in coronary blood flow. Additionally, risk factors such as hypertension and diabetes can exacerbate endothelial damage, promoting further plaque formation. Clinically, this manifests as angina, myocardial infarction, or heart failure, depending on the severity and rapidity of coronary obstruction. Understanding these anatomical and pathological changes is crucial for diagnosis and management.
9	The pterygoid venous plexus holds clinical significance due to its connections and location. Situated around the pterygoid muscles, it is connected to the cavernous sinus via the emissary veins, which can act as a route for the spread of infections from the face to the cavernous sinus, potentially leading to serious complications like cavernous sinus thrombosis. Additionally, its proximity to the maxillary artery and branches of the trigeminal nerve makes it relevant in surgical interventions and anesthetic procedures in the maxillofacial region.
10	Calot's triangle, also known as the cystohepatic triangle, is an anatomical space of significant clinical importance in gallbladder surgery. It is bordered by the cystic duct, the common hepatic duct, and the inferior edge of the liver. The critical view of safety in laparoscopic cholecystectomy involves clear identification of this triangle to safely isolate and divide the cystic duct and artery, reducing the risk of injury to the common bile duct and nearby vascular structures, which can be life-threatening if damaged.
11	To illustrate the transverse sections of the pons at both the upper and lower levels, I'll describe each section and then create a labeled image for you. Upper Level of Pons: Basis Pontis: This is the ventral portion and contains many transversely running fibers. Tegmentum: Located dorsally, it includes ascending sensory and descending motor tracts. Pontine Nuclei: Scattered throughout the basis pontis, these are involved in relaying signals from the forebrain to the cerebellum. Cranial Nerve Nuclei: Such as the trigeminal nerve nucleus. Lower Level of Pons: Basis Pontis: Similar to the upper level, with transverse fibers prominently visible. Tegmentum: Contains longitudinally oriented fibers and nuclei. Facial Nerve Fibers: These fibers loop around the abducens nucleus, forming the facial colliculus. Abducens Nucleus: This is more prominent at this level. I'll create a labeled diagram showing these features for both levels. Let's start with that. Here are the labeled illustrations of the transverse sections of the pons at the upper and lower levels. Each section is clearly marked to show the relevant anatomical features. You can view and examine the details in the images provided (Figure 2).
12	Basal Ganglia Parts of the Basal Ganglia: Caudate Nucleus Putamen Globus Pallidus (divided into internal and external segments) Subthalamic Nucleus Substantia Nigra (divided into pars compacta and pars reticulata) Major Connections of the Basal Ganglia: Input: Primarily from the cortex, projecting to the striatum (caudate and putamen) and the subthalamic nucleus. Output: From the globus pallidus internal segment (GPi) and the substantia nigra pars reticulata (SNr) to the thalamus, which then projects back to the cortex. Intrinsic Connections: Include connections between the caudate and putamen to the globus pallidus and substantia nigra. The subthalamic nucleus sends excitatory inputs to the GPi/SNr and receives inhibitory feedback from these regions. Limbic Lobe Parts of the Limbic Lobe: Cingulate Gyrus Parahippocampal Gyrus Hippocampus Dentate Gyrus Amygdala Olfactory Cortex Major Connections of the Limbic Lobe: Hippocampal Formation: Receives inputs from various cortical areas and sends outputs through the fornix to structures such as the mammillary bodies of the hypothalamus, and the thalamus. Amygdala: Connected to the hippocampus, thalamus, hypothalamus, septal areas, and prefrontal cortex, playing a key role in emotion regulation and memory. Cingulate Gyrus: Receives inputs from the thalamus and connects to the hippocampus and other cortical areas, involved in emotional processing and executive function. Parahippocampal Gyrus: Links the entorhinal cortex to the hippocampus, critical for memory encoding and retrieval. These systems are intricately linked and play crucial roles in motor control, emotional regulation, and memory processing.
13	Muscular tissue is classified into three main types: skeletal, cardiac, and smooth muscle. Each type has distinct features at the ultrastructural level, which enable their specific functions. Here's an overview of their ultrastructures along with a diagram to illustrate these differences. Ultrastructure of Skeletal Muscle Sarcolemma: The plasma membrane of a muscle fiber that encloses its cellular contents. Sarcoplasmic Reticulum (SR): A specialized form of endoplasmic reticulum that stores and releases calcium ions, crucial for muscle contraction. Myofibrils: Long, rod-like units within muscle fibers, consisting of repeating sections called sarcomeres, the functional units of muscle contraction. Sarcomeres: Contain the thin (actin) and thick (myosin) filaments whose interaction leads to muscle contraction. They are delineated by Z-discs. T-Tubules: Extensions of the sarcolemma that penetrate into the cell's interior and help propagate the action potential to ensure that the contraction is coordinated. Ultrastructure of Cardiac Muscle Intercalated Discs: Specialized connections between cardiac muscle cells that facilitate the synchronized contraction of the heart muscle. Mitochondria: Abundant in cardiac muscle cells to meet high energy demands. Myofibrils: Similar to skeletal muscle but with some differences in the arrangement for continuous, rhythmic contraction. Sarcolemma, SR, and T-Tubules: Similar function as in skeletal muscle but adapted for the heart’s continuous activity. Ultrastructure of Smooth Muscle Spindle-Shaped Cells: Each cell has a single nucleus and lacks the striations seen in skeletal and cardiac muscle. Dense Bodies: Analogous to Z-discs, these are attachment sites for actin filaments within the cell. Caveolae: Small invaginations in the sarcolemma that function similarly to T-tubules in skeletal and cardiac muscle. Less Developed Sarcoplasmic Reticulum: Relies more on extracellular calcium for contractions. Now, let's create a diagram that illustrates these features for each type of muscle tissue. Here is the diagram illustrating the ultrastructure of three types of muscular tissues: skeletal, cardiac, and smooth muscle. Each type is depicted with its specific structures and is labeled for clarity. You can examine the features of each muscle type in detail in the image provided (Figure 3).
14	The Lyon hypothesis, also known as Lyonization, is a fundamental concept in genetics formulated by geneticist Mary Frances Lyon in 1961. It explains the mechanism of X-chromosome inactivation in female mammals, which is a process that balances the expression of X-chromosome genes between males and females. Key Aspects of the Lyon Hypothesis: Random X-Chromosome Inactivation: In female mammals, one of the two X chromosomes in each cell is randomly inactivated during early embryonic development. This means each cell 'chooses' either the maternal or paternal X chromosome to silence, leading to a mosaic of cells expressing genes from either the maternal or paternal X chromosome. Barr Body Formation: The inactivated X chromosome condenses into a dense structure known as a Barr body, which is generally located at the periphery of the nucleus. This structure is largely transcriptionally inactive, meaning it does not express most of its genes. Maintenance of Inactivation: Once an X chromosome is inactivated in a particular cell, the same chromosome remains inactive in all descendant cells of that cell line. This heritable form of gene regulation ensures that the pattern of inactivation is maintained throughout the life of the individual. Exceptions and Escapees: Some genes on the inactivated X chromosome can escape inactivation and remain active. The number and identity of these escape genes can vary among different species and even among individuals. Functional Dosage Compensation: The rationale behind X-chromosome inactivation is to achieve dosage compensation between males (who have one X chromosome) and females (who have two). Without this mechanism, females would express double the amount of proteins encoded by X-linked genes, potentially leading to harmful effects. The Lyon hypothesis has been fundamental in understanding not only X-chromosome inactivation but also broader aspects of epigenetics and gene regulation. It has implications for the study of diseases linked to the X chromosome, as variations in the pattern or extent of X inactivation can lead to conditions such as X-linked disorders in females.
15	Cleavage and the formation of the blastocyst are crucial early steps in the development of an embryo. Here’s a detailed look at these processes: Cleavage Cleavage is the series of rapid mitotic cell divisions that an embryo undergoes after fertilization. During cleavage, the zygote (fertilized egg) divides into smaller cells called blastomeres without increasing in overall size. This division happens very quickly and is characterized by the following: Rapid Cell Division: The initial divisions occur in quick succession. Each division reduces the size of individual cells, while the total volume of the embryo remains constant. Compaction: As the cells divide, they begin to tightly pack together, a process known as compaction. This ensures that the cells maximize their contact and communication, which is essential for the next stages of development. Syncytial Stage: In some organisms like insects, this stage involves nuclei dividing without cell division, forming a multinucleate cell. Formation of the Blastocyst Following cleavage, the embryo becomes a morula, which is a solid ball of cells. This structure then undergoes further development to form the blastocyst, typically around five to six days after fertilization in humans. The formation of the blastocyst involves: Blastulation: This is the process by which the morula develops a fluid-filled cavity called the blastocoel, transforming it into a blastocyst. The blastocyst consists of a sphere with a fluid-filled inner cavity. Differentiation of Cells: The cells of the blastocyst differentiate into two main types: Inner Cell Mass (ICM): A cluster of cells inside the blastocyst that will develop into the fetus. Trophectoderm: The outer layer of cells that will form part of the placenta and provide nutrients to the developing embryo. Hatching: The blastocyst eventually "hatches" from the zona pellucida, the glycoprotein shell surrounding the embryo, allowing for implantation into the uterine wall. Implantation The blastocyst implants in the endometrium of the uterus. This is facilitated by interactions between the cells of the trophectoderm and the endometrial lining. Successful implantation is critical for the continued development of the embryo and the establishment of pregnancy. These processes — from cleavage through the formation of the blastocyst and its implantation — are critical for establishing a viable pregnancy and lay the foundation for all subsequent development.
16	Certainly! Below are five multiple-choice questions (MCQs) designed to assess MBBS students on competency number AN 10.3, which involves the brachial plexus. Each question includes a correct answer and an explanation. Question 1 What are the roots of the brachial plexus? A) C5-T1 B) C1-C5 C) T1-T5 D) C3-C8 Correct Answer: A) C5-T1 Explanation: The brachial plexus is formed by the ventral rami of the fifth through the first thoracic spinal nerves (C5-T1). This is crucial for understanding the origin and initial nerve contributions that form the plexus. Question 2 Which muscle is predominantly supplied by the musculocutaneous nerve, a branch of the brachial plexus? A) Biceps Brachii B) Triceps Brachii C) Pectoralis Major D) Latissimus Dorsi Correct Answer: A) Biceps Brachii Explanation: The musculocutaneous nerve, emerging from the lateral cord of the brachial plexus, primarily innervates the biceps brachii muscle. This illustrates the functional distribution of nerves within the brachial plexus. Question 3 The axillary nerve, a branch of the brachial plexus, is closely related to which anatomical structure? A) Axillary artery B) Carotid artery C) Radial artery D) Femoral artery Correct Answer: A) Axillary artery Explanation: The axillary nerve arises from the posterior cord of the brachial plexus and travels along the axillary artery. It is important to note the relationship between major nerves and vessels for surgical and diagnostic purposes. Question 4 Which branch of the brachial plexus is primarily responsible for the sensory innervation of the thumb? A) Ulnar nerve B) Radial nerve C) Median nerve D) Axillary nerve Correct Answer: C) Median nerve Explanation: The median nerve, which branches from both the medial and lateral cords of the brachial plexus, provides sensory innervation to the thumb. Knowledge of sensory distributions is vital for diagnosing nerve injuries. Question 5 What is the course of the radial nerve in relation to the humerus? A) Medial to the humerus B) Lateral to the humerus C) Posterior in the radial groove of the humerus D) Anterior to the humerus Correct Answer: C) Posterior in the radial groove of the humerus Explanation: The radial nerve runs in the radial groove on the posterior aspect of the humerus. This specific anatomical relationship is critical for understanding the potential sites for nerve compression or injury. These questions cover key aspects of the brachial plexus, including formation, functional branches, anatomical relationships, and clinical relevance, providing a comprehensive assessment tool for the specified competency.
17	Certainly! Here are five True or False questions designed to assess MBBS students on competency number AN 15.3, which focuses on the anatomy of the femoral triangle. Each question is followed by the correct answer and a brief explanation. Question 1 True or False: The inguinal ligament forms the base of the femoral triangle. Answer: False Explanation: The inguinal ligament actually forms the superior boundary of the femoral triangle, not the base. The base of the triangle is formed by the medial border of the adductor longus muscle. Question 2 True or False: The femoral nerve is located medial to the femoral artery within the femoral triangle. Answer: False Explanation: The femoral nerve is actually located lateral to the femoral artery within the femoral triangle. This positioning is important for surgical interventions and regional anesthesia. Question 3 True or False: The sartorius muscle forms the lateral border of the femoral triangle. Answer: True Explanation: The sartorius muscle indeed forms the lateral border of the femoral triangle. This muscle's positioning helps in identifying the boundaries of the triangle during anatomical dissections and examinations. Question 4 True or False: The roof of the femoral triangle is formed by the fascia lata. Answer: True Explanation: The roof of the femoral triangle is formed by the fascia lata, specifically by the cribriform fascia, which is an area where the fascia lata is pierced by saphenous vein and other structures. Question 5 True or False: The adductor longus and the adductor magnus form the floor of the femoral triangle. Answer: False Explanation: Only the adductor longus contributes to forming the floor of the femoral triangle along with the pectineus muscle. The adductor magnus is not involved in the floor of the femoral triangle but is located more posteriorly. These questions help evaluate the student's understanding of the anatomical features and clinical significance of the femoral triangle.
18	Here are five matching questions designed to assess MBBS students on competency number AN 18.4, which involves detailed knowledge of the knee joint. Each question is followed by a set of options that the students need to match correctly. Answers and explanations are provided for clarity. Question 1: Match the type of joint to the correct description. Options: A. Hinge joint B. Ball and socket joint C. Saddle joint Statements: Permits primarily flexion and extension — Allows movement in multiple planes, including rotation — Supports movements in two planes but not rotation — Answers: A - Hinge joint: The knee joint is a modified hinge joint that primarily allows for flexion and extension, with limited rotation when the knee is flexed. B - Ball and socket joint: This description fits joints like the hip or shoulder, not the knee. C - Saddle joint: Typical of the thumb base but not applicable to the knee. Question 2: Match the ligament with its primary function or location. Options: A. Anterior cruciate ligament (ACL) B. Posterior cruciate ligament (PCL) C. Medial collateral ligament (MCL) D. Lateral collateral ligament (LCL) Statements: Prevents posterior displacement of the tibia on the femur — Resists valgus stress at the knee — Prevents anterior displacement of the tibia on the femur — Resists varus stress at the knee — Answers: B - Posterior cruciate ligament (PCL): It prevents the femur from sliding off the front of the tibia and from the tibia sliding backward on the femur. C - Medial collateral ligament (MCL): It provides stability against inward forces. A - Anterior cruciate ligament (ACL): It primarily prevents the tibia from sliding out in front of the femur. D - Lateral collateral ligament (LCL): It provides stability against outward forces. Question 3: Match the muscle to its action on the knee joint. Options: A. Quadriceps B. Hamstrings C. Gastrocnemius Statements: Extension of the knee — Flexion of the knee — Assists in knee flexion — Answers: A - Quadriceps: This group of muscles is primarily responsible for knee extension. B - Hamstrings: These muscles are primarily involved in knee flexion. C - Gastrocnemius: While primarily a calf muscle, it assists in bending the knee. Question 4: Match the knee joint structure to its description. Options: A. Synovial membrane B. Menisci C. Bursae Statements: Fibrocartilaginous structures that deepen the joint surface — Fluid-filled sacs that reduce friction — Lines the joint capsule and secretes synovial fluid — Answers: B - Menisci: They act as shock absorbers and deepen the joint surface to aid in stability. C - Bursae: These help to cushion and reduce friction between tissues of the body. A - Synovial membrane: It lines the joint capsule, secreting synovial fluid to lubricate the joint. Question 5: Match the nerve to its function in the knee joint. Options: A. Femoral nerve B. Sciatic nerve C. Tibial nerve Statements: Provides sensation to the anterior and medial aspects of the knee — Provides sensation to the posterior aspect of the knee — Contributes to motor control over muscles that flex the knee — Answers: A - Femoral nerve: It innervates the anterior portion of the thigh and mediates sensations from the anterior and medial knee. C - Tibial nerve: Part of the sciatic nerve, it provides sensory input from the posterior knee. B - Sciatic nerve: It primarily controls the hamstrings, which flex the knee. These questions test the students’ understanding of various structural and functional aspects of the knee joint, ensuring a comprehensive evaluation of their knowledge.
19	Certainly! Here's a short answer question designed to assess knowledge related to competency number AN 24.3, which involves understanding the structure and function of a bronchopulmonary segment. Question: Describe the anatomical structure and functional significance of a bronchopulmonary segment. Answer: A bronchopulmonary segment is a specific, functionally independent region of the lung, which has its own segmental bronchus and accompanying branches of the pulmonary artery and veins. Each segment is pyramidal in shape, with its apex pointing towards the root of the lung and its base facing the surface of the lung. These segments are separated from each other by connective tissue septa, which help contain infections or pathologies within a segment, limiting their spread to other parts of the lung. Functionally, bronchopulmonary segments allow for the localized treatment of lung diseases. For example, surgical resection of a segment may be performed without significantly affecting adjacent segments. Each segment's independent blood supply and airway system facilitate such targeted interventions, making them critical units for pulmonary surgery and diagnosis. This segmentation also enhances the efficiency of gas exchange by maintaining organized airflow and blood flow within each segment.
20	Certainly! Here’s a case-based multiple-choice question (MCQ) designed to assess MBBS students on competency number AN 58.4, which focuses on the anatomical knowledge relevant to lateral medullary syndrome. Case Scenario: A 55-year-old male presents to the emergency department with sudden onset of vertigo, nausea, and vomiting. He also reports difficulty swallowing and a feeling of numbness on the right side of his face and the left side of his body. His medical history includes chronic hypertension and smoking. Physical examination reveals a loss of pain and temperature sensation on the contralateral side of the body and ipsilateral Horner’s syndrome. Question: Based on the symptoms described in the case, which artery is most likely involved in the development of this patient's condition? A) Anterior inferior cerebellar artery (AICA) B) Posterior inferior cerebellar artery (PICA) C) Middle cerebral artery (MCA) D) Basilar artery Correct Answer: B) Posterior inferior cerebellar artery (PICA) Explanation: The patient’s symptoms are indicative of lateral medullary syndrome, also known as Wallenberg’s syndrome, which is typically caused by occlusion of the posterior inferior cerebellar artery (PICA). This syndrome affects the lateral part of the medulla oblongata where PICA provides blood supply. Key symptoms and their anatomical correlations include: Vertigo, nausea, and vomiting: These symptoms arise due to involvement of the vestibular nuclei. Difficulty swallowing and hoarseness: Resulting from damage to the nucleus ambiguus, which affects the vagus nerve components. Ipsilateral loss of pain and temperature sensation on the face: Due to impairment of the spinal trigeminal nucleus. Contralateral loss of pain and temperature sensation on the body: Stemming from damage to the spinothalamic tract. Ipsilateral Horner’s syndrome: This occurs due to disruption of the descending sympathetic fibers. Options A, C, and D are less likely as they involve different regions or present with different clinical manifestations. The middle cerebral artery (MCA) affects lateral aspects of the cerebral hemisphere and presents with motor and sensory deficits, whereas the basilar artery would lead to brainstem signs that are more central and involve both sides of the body. AICA involvement might cause similar symptoms but typically affects structures associated with the cerebellum more directly, often leading to more pronounced coordination deficits.
21	Here are five structured oral viva voce questions designed to assess MBBS students on competency number AN 63.1, which involves detailed anatomical knowledge of the third, fourth, and lateral ventricles of the brain. Each question is aimed at testing the understanding of the parts, boundaries, and features of these ventricles. Question 1: Describe the boundaries and connections of the lateral ventricles. Expected Answer: The lateral ventricles, located in each hemisphere of the brain, consist of a frontal (anterior) horn, body, atrium, occipital (posterior) horn, and temporal (inferior) horn. The frontal horn is bounded by the corpus callosum above and the caudate nucleus below. The body extends posteriorly and is bordered by the corpus callosum above and thalamus below. The atrium is where the body, posterior horn, and inferior horn meet. The posterior horn extends into the occipital lobe, while the inferior horn extends into the temporal lobe. The lateral ventricles communicate with the third ventricle via the interventricular foramen (of Monro). Question 2: What are the features and functions of the third ventricle? Expected Answer: The third ventricle is a narrow, vertically oriented cavity located between the two halves of the thalamus. Its boundaries include the thalamus and hypothalamus on either side. Anteriorly, it is bounded by the lamina terminalis, and it connects to the fourth ventricle via the cerebral aqueduct. The third ventricle houses cerebrospinal fluid (CSF) and communicates with the lateral ventricles through the interventricular foramen. Functionally, it is part of the ventricular system that cushions the brain, removes metabolic waste, and provides a stable chemical environment. Question 3: Explain the anatomy of the fourth ventricle and its clinical significance. Expected Answer: The fourth ventricle is located at the hindbrain, posterior to the pons and upper half of the medulla, and anterior to the cerebellum. It resembles a tent shape with a roof formed by the cerebellum and a floor by the dorsal surface of the pons and upper medulla. The ventricle communicates with the third ventricle through the cerebral aqueduct and with the subarachnoid space via the lateral apertures (foramina of Luschka) and a median aperture (foramen of Magendie). Clinically, obstruction of these pathways, as seen in conditions like hydrocephalus, can lead to increased intracranial pressure and associated neurological symptoms. Question 4: How is the choroid plexus associated with the lateral ventricles, and what is its role? Expected Answer: The choroid plexus is found in the body, atrium, and inferior horn of each lateral ventricle, as well as in the roof of the third and fourth ventricles. It consists of a layer of ependymal cells and vascularized connective tissue that produces cerebrospinal fluid (CSF). This CSF production is crucial for maintaining the buoyancy and protection of the brain, as well as for the removal of metabolic wastes. Question 5: Detail the path and flow of cerebrospinal fluid (CSF) through the ventricular system, starting from the lateral ventricles. Expected Answer: CSF is produced mainly by the choroid plexus in the lateral ventricles. From here, it flows through the interventricular foramen into the third ventricle, where additional CSF is added by its choroid plexus. CSF then travels down the cerebral aqueduct to the fourth ventricle, where again, more CSF is added by the choroid plexus. From the fourth ventricle, CSF flows through the lateral and median apertures into the subarachnoid space surrounding the brain and spinal cord, and finally, it is absorbed into the venous system via the arachnoid granulations. These questions comprehensively cover the anatomy, function, and clinical relevance of the ventricular system, ensuring a thorough assessment of the student's understanding.
22	Certainly! Here are five fill-in-the-blank questions aimed at assessing MBBS students for NMC competency AN 38.1, which requires understanding the morphology, structure, nerve supply, blood supply, and actions of the intrinsic and extrinsic muscles of the larynx. Question 1 The ______ muscle is responsible for adducting the vocal cords, and its action is crucial for phonation. Answer: Lateral cricoarytenoid Question 2 All intrinsic muscles of the larynx are innervated by the ______ nerve except for the cricothyroid muscle, which is innervated by the external branch of the superior laryngeal nerve. Answer: Recurrent laryngeal Question 3 The ______ muscle elevates the larynx during swallowing and is considered an extrinsic muscle of the larynx. Answer: Thyrohyoid Question 4 The major blood supply to the larynx is provided by the ______ artery and ______ artery, branches of the thyroid artery. Answer: Superior laryngeal; inferior laryngeal Question 5 The ______ muscle, an intrinsic muscle of the larynx, functions to tense the vocal cords, thus increasing the pitch of the voice. Answer: Cricothyroid These questions comprehensively cover various aspects of the anatomy and physiology of the larynx, targeting the specific details required by the competency standard.
23	Assessing the competency of MBBS students in identifying and describing the course and branches of important blood vessels and nerves in the hand (competency number AN 12.7) requires a combination of theoretical knowledge and practical skills. Here are several effective methods to evaluate this competency: 1. Written Examinations Multiple Choice Questions (MCQs): These can test students on specific details about the anatomy of the hand, such as naming blood vessels and nerves, and their specific branches and courses. Short Answer Questions: Prompt students to describe specific structures and their anatomical relations or functions in a concise manner. 2. Practical Examinations Cadaveric Dissection: Direct dissection provides a hands-on method for students to identify and explore the anatomy of the hand. Assessments can be made on their ability to correctly identify structures and demonstrate knowledge of their functional relevance. Annotated Diagrams: Students can be asked to label or draw detailed diagrams of the hand’s vascular and nervous systems, demonstrating their understanding of the spatial relationships and branches. 3. Objective Structured Clinical Examinations (OSCEs) Identification Station: Use prosections or models where students must identify tagged structures. This tests their quick recall and understanding of the anatomical layout. Viva Voce: Oral questioning around a prosection or model, where students describe what they see and explain the function of various blood vessels and nerves. 4. Imaging Interpretation Radiographic Analysis: Provide X-ray, MRI, or ultrasound images where students must identify and trace the course of nerves and vessels in the hand. This assesses their ability to apply anatomical knowledge to clinical imaging. 5. Clinical Scenario Simulations Case Studies: Present clinical scenarios involving injuries or diseases of the hand’s vascular and nerve structures. Students must diagnose based on symptoms, suggest treatments, and discuss implications of damage to these structures. Problem-Based Learning: Use hypothetical but realistic problems where students must apply their knowledge of hand anatomy in a clinical context, encouraging integration of anatomical knowledge with clinical reasoning. 6. Peer Teaching Peer Assessment: Students teach a segment of the course content to peers, such as the anatomy of the hand’s nervous system, and then receive feedback on their accuracy, clarity, and teaching method. 7. Self-Assessment Reflective Journals: Students maintain a journal of what they have learned about the hand's anatomy, reflecting on difficulties they encountered and how they overcame them. This encourages self-directed learning and retention of anatomical details. Each of these methods targets different aspects of learning and allows for a comprehensive assessment of the student’s mastery of the anatomy of the hand, ensuring a well-rounded evaluation of their competency.
24	Conducting a DOAP (Demonstration-Observation-Assessment-Participation) session is an effective method for teaching and assessing MBBS students, especially for complex anatomical competencies like AN 33.3, which involves the temporomandibular joint (TMJ). Here’s how to organize and execute such a session step-by-step: Step 1: Preparation Educational Materials: Prepare detailed visuals, models, and possibly video demonstrations of the TMJ to help in explaining its structure and functions. Resource Allocation: Ensure access to anatomical models or cadavers, diagrams, and radiographic images to demonstrate the TMJ's anatomy. Session Objectives: Clearly define the learning objectives. For this session, these might include understanding the articulating surfaces, joint type, and movements facilitated by the TMJ. Step 2: Demonstration Instructor-Led Demonstration: Begin with a detailed explanation using models or diagrams. Highlight the articulating surfaces (the temporal bone and the mandibular condyle), the type of joint (a modified hinge type synovial joint that allows for hinge and sliding movements), and the specific movements (elevation, depression, protrusion, retraction, and lateral movements). Video Demonstration: Show a video that details the movements of the TMJ and its functional relevance to activities such as chewing and speaking. Step 3: Observation Peer Observation: Have students observe each other identifying the TMJ on a model or skull. Encourage them to discuss the articulating surfaces and possible movements. Radiographic Analysis: Provide X-rays or MRI scans showing the TMJ in different positions and ask students to identify structures and movement limitations. Step 4: Assessment Question and Answer: Conduct a brief quiz or oral questioning to assess students' understanding of the joint's anatomy and mechanics. Practical Test: Students could be asked to demonstrate on a model or diagram the movements of the TMJ and discuss clinical scenarios where TMJ disorders might affect these movements. Step 5: Participation Hands-on Practice: Allow students to manipulate models or software simulations to explore the TMJ’s anatomy. Role Play: Students can perform role-plays that involve diagnosing TMJ disorders, discussing symptoms, and proposing initial management steps. Feedback Session: Conclude with a feedback session where students share what they learned and instructors provide corrections or additional insights. Step 6: Reflection Reflective Learning: Encourage students to write a short reflection on what they learned about the TMJ and how they can apply this knowledge clinically. Feedback for Improvement: Gather student feedback on the DOAP session to improve future sessions. Step 7: Follow-Up Supplementary Materials: Provide additional reading materials or online resources for students who wish to delve deeper into the subject. Continuous Assessment: Integrate assessment elements from this session into subsequent practical or theoretical exams. This structured approach not only deepens understanding but also enhances the practical skills of students, preparing them for clinical applications of their anatomical knowledge.
25	Yes, AETCOM, which stands for "Attitude, Ethics, and Communication," is a module introduced by the National Medical Commission (NMC) of India for MBBS students. It's designed to develop the essential competencies in communication, ethics, and professionalism required for medical practice. The AETCOM module spans the entire duration of the MBBS course and is integrated into various stages of the curriculum to ensure that students not only learn medical science but also understand the ethical, legal, and social aspects of healthcare. The module is structured to be taught in different phases of the MBBS program: Phase I (MBBS Professional-1): Focuses on the student's transition into medical education, emphasizing communication with peers and faculty, and introducing basic ethical concepts relevant to medical students. Phase II and III: Expands on these foundations with a focus on doctor-patient communication, informed consent, confidentiality, and ethical decision-making in clinical practice. Internship: Applies these concepts in real-world clinical settings, reinforcing ethical practice, effective communication, and professionalism. AETCOM aims to cultivate a more holistic approach to medical education by integrating these competencies early and throughout medical training, preparing students not just as clinicians but as empathetic communicators and ethical decision-makers.
26	Conducting an introductory session for AETCOM module 1.5, focusing on demonstrating respect and following the correct procedures when handling cadavers and other biological tissues, is crucial for instilling ethical and respectful practices in medical students. Here’s a structured approach for a two-hour session: Step 1: Preparation Materials Needed: Prepare educational materials including presentation slides, videos, protocol handouts, and relevant ethical guidelines. Environment Setup: Arrange a suitable learning environment, preferably a lecture hall or a demonstration room that accommodates a respectful discussion about handling cadavers. Expert Involvement: Involve an ethicist or a senior anatomist to discuss ethical considerations and technical handling. Step 2: Introduction (15 minutes) Welcome and Objectives: Begin with a brief introduction, outlining the session's objectives and its importance in medical education. Background Information: Provide a brief history of the use of cadavers in medical science, emphasizing the ethical considerations and respect that have evolved over time. Step 3: Ethical and Legal Considerations (30 minutes) Presentation: Use a PowerPoint presentation to discuss the ethical and legal aspects of using human cadavers for medical training. Include topics like consent, the dignity of the donor, and confidentiality. Discussion: Encourage an open discussion about the emotional impact and ethical responsibility involved in handling cadavers. Step 4: Demonstrating Procedures (30 minutes) Video Demonstration: Show a video detailing the proper procedures for handling, preserving, and studying cadavers. Highlight the importance of using personal protective equipment (PPE) and following standard operating procedures. Practical Guidelines: Discuss the step-by-step procedures for entering the anatomy lab, the demeanor expected, and the respectful treatment of cadavers. Step 5: Role-Playing and Scenario-Based Learning (30 minutes) Role-Playing: Conduct role-playing exercises where students practice entering the dissection lab and interacting with cadavers under supervision. Focus on the respectful handling and procedural aspects. Scenarios: Present hypothetical scenarios involving ethical dilemmas in handling cadavers and ask students how they would respond, fostering critical thinking about ethics in practice. Step 6: Reflection and Discussion (10 minutes) Group Reflection: Allow students to share their feelings and thoughts about handling cadavers for the first time. Discuss any concerns and how they think they should manage these feelings professionally. Feedback on the session: Gather feedback from students on the clarity of the ethical guidelines provided and their comfort level with the procedures demonstrated. Step 7: Conclusion and Further Resources (5 minutes) Closing Remarks: Emphasize the importance of dignity and respect in all medical practices, especially in dealing with cadavers. Further Reading: Provide students with additional resources, articles, and reading materials on ethical considerations and the history of anatomical studies. Additional Tips Continuous Learning: Remind students that respect for cadavers and adherence to ethical procedures is an ongoing process throughout their medical education. Support System: Inform students about the support systems available if they experience discomfort or ethical dilemmas during their anatomy studies. This structured session aims to ensure that MBBS students understand and respect the profound responsibility involved in handling cadavers, setting a foundation for ethical medical practice.
27	Certainly! Below is a detailed outline for a PowerPoint presentation on the formation of the rectus sheath and its contents, suitable for competency number AN 44.3. This outline includes suggested slides and key points to cover, ensuring a comprehensive overview. PowerPoint Presentation: Formation of the Rectus Sheath and Its Contents Slide 1: Title Slide Title: Formation of the Rectus Sheath and Its Contents Subtitle: Understanding Abdominal Anatomy Presenter’s Name and Date Course/Session Title Slide 2: Introduction to Rectus Sheath Definition: Explain what the rectus sheath is. Importance: Discuss its role in the abdominal wall structure and protection of abdominal contents. Slide 3: Anatomical Position of the Rectus Sheath Location: Describe its position in the abdominal wall. Components: Briefly introduce the structures that form the sheath. Slide 4: Formation of the Rectus Sheath Layers Contributing to the Sheath: Anterior layer Posterior layer Fascial Layers: Explain the role of the external oblique, internal oblique, and transversus abdominis in forming these layers. Slide 5: Detailed Formation Process Above the Arcuate Line: Describe how the anterior layer is formed by the aponeuroses of the external and half the internal oblique. Outline how the posterior layer includes the aponeuroses of the other half of the internal oblique and the transversus abdominis. Below the Arcuate Line: Detail how all three muscle aponeuroses pass anterior to the rectus abdominis, leaving the rectus muscle only covered by the transversalis fascia and peritoneum posteriorly. Slide 6: Contents of the Rectus Sheath Rectus Abdominis Muscle: Main occupant. Blood Vessels: Superior and inferior epigastric arteries and veins. Nerves: Segmental thoracoabdominal nerves (T7-T11) and subcostal nerve (T12). Slide 7: Clinical Significance Surgical Access: Importance in abdominal surgeries. Hernias: Potential sites for hernia development, especially near the arcuate line. Blood Supply: Relevance in flap surgeries and recovery. Slide 8: High-Resolution Images Anatomical Diagrams: Show detailed labeled diagrams of the rectus sheath formation. Cross-Sectional Views: Display cross-sectional anatomy to visualize the sheath's formation above and below the arcuate line. Slide 9: Case Studies / Clinical Correlations Hernia Cases: Discuss cases where understanding the rectus sheath anatomy was crucial. Surgical Repair Techniques: Examples of how this anatomical knowledge aids in effective surgical planning and execution. Slide 10: Summary Recap the key points about the formation and importance of the rectus sheath. Highlight the educational objectives achieved in the presentation. Slide 11: Questions and Discussion Invite questions from the audience. Provide your contact information for further queries or follow-up. Slide 12: References and Further Reading List textbooks, articles, and online resources for students to explore more about the rectus sheath. This structure ensures that the presentation covers all essential aspects of the rectus sheath in a clear and educational manner, making it ideal for teaching MBBS students. The inclusion of high-resolution images and clinical correlations will enhance understanding and engagement.
28	I can definitely help evaluate a written answer by providing feedback on its accuracy, completeness, clarity, and relevance to the question asked. However, I can't assign specific grades or marks like a human instructor might, since I don't have access to your grading rubric or specific educational standards. But I can certainly assist in assessing whether the answer covers key points required by the question and suggest improvements or identify areas that may need additional information. If you have a written answer you'd like feedback on, feel free to share it!
29	The student's answer to the question "Describe the arterial supply of the uterus" is brief and partially correct but lacks detail. Here’s a breakdown of the feedback and a potential mark out of 5: Feedback: Accuracy: The student correctly identifies the main arteries supplying the uterus: the uterine artery and the ovarian artery. This is fundamentally correct. Completeness: The answer is very brief and lacks detail. It would be beneficial to expand on how these arteries contribute to the blood supply of the uterus. For instance, explaining that the uterine artery is the primary source of blood to the uterus, arising from the internal iliac artery, and detailing its branches (e.g., the ascending, transverse, and descending branches) would provide a more complete picture. Similarly, mentioning how the ovarian artery supplements the blood supply to the uterus, especially the fundus, through anastomoses with branches of the uterine artery, would enhance the answer. Clarity: While the answer is clear and straightforward, the simplicity also makes it superficial. Adding specific details about the arterial branches and their specific regions of supply within the uterus would improve clarity and understanding. Relevance: The answer is relevant to the question but lacks depth, which is necessary for a thorough understanding in a medical context. Suggested Mark: Given the correctness but incomplete nature of the response, I would suggest a score of 2 out of 5. This score reflects that the student has basic knowledge but failed to provide a detailed description as likely expected in a medical curriculum context. Recommendations for Improvement: The student should aim to include: Details of the branches of the uterine artery and their specific areas of supply within the uterus. An explanation of the role of the ovarian artery and its anastomoses with the uterine artery. Possible inclusion of any clinical relevance, such as the importance of these arteries in conditions like fibroids or during procedures like hysterectomy. Providing these additional details would result in a more comprehensive answer that demonstrates a deeper understanding of the topic.
30	The image you provided appears to be a sketch of the brain's ventricular system viewed from above, focusing specifically on the cerebellum and brainstem area (Figure 4). Key features in the sketch: The large, butterfly or "H"-shaped structure in the center likely represents the cerebellum. The small circular area at the center where the two parts of the "H" meet could represent the fourth ventricle. The linear structures extending upward from the center towards the top of the image might be indicating the cerebral aqueduct leading into the third ventricle, though this specific transition isn't clear in the sketch. This is a simplistic representation, and some anatomical details may not be fully accurate or detailed enough for precise identification beyond these general structures. If there's a specific part of the sketch you're curious about or need more detailed information on, feel free to point it out!
31	The image you've provided appears to be a simple line drawing of a human stomach. The structure is recognizable by its shape, which includes the wider fundus at the top, the body, and the narrower pylorus at the bottom leading into the small curve of the duodenum. (Figure 5).
32	The red arrow in the image points to the central portion of the stomach, known as the body of the stomach. The body is the main, central region of the stomach between the fundus above and the pyloric part below. It functions primarily as the site for mixing and digestion of food using gastric juices, which are secreted from the stomach lining (Figure 6).
33	To get accurate, reliable, and valid answers from a conversational AI like me, especially in the context of improving anatomy education, it's beneficial to employ specific strategies. Here are some techniques to consider: 1. Provide Clear and Specific Questions Detail is Key: The more specific the question, the more accurate and tailored the response can be. Include details about the context or particular aspect of anatomy you're interested in. 2. Utilize Follow-Up Questions Clarify and Refine: If an initial answer isn’t fully satisfactory or seems incomplete, ask follow-up questions to narrow down or expand on the information provided. 3. Employ Iterative Questioning Layered Learning: Start with broad questions and progressively narrow down to more detailed aspects. This helps in building a comprehensive understanding and checks for consistency in the responses. 4. Cross-Verify Answers Use Multiple Sources: After receiving an answer, it can be beneficial to cross-verify the information with textbooks, peer-reviewed articles, or trusted online medical resources. This practice enhances reliability and encourages critical thinking. 5. Request for Diagrams and Visuals Visual Aids: Since anatomy is a visually intensive subject, request diagrams or illustrations to accompany explanations. Visuals can help clarify complex structures and spatial relationships. 6. Integration of Interactive Learning Tools Interactive Quizzes: Ask for quizzes or practice questions on specific anatomy topics to test understanding and retention. Scenario-Based Learning: Request explanations or discussions based on hypothetical clinical scenarios that apply anatomical knowledge practically. 7. Specify the Level of Detail Adjust Complexity: Indicate whether you need a brief overview or a detailed explanation. This helps tailor the responses to suit beginner level learners or advanced students. 8. Incorporate Terminology Checks Define Terms: When discussing less familiar or complex topics, ask for definitions or simplifications of medical terms used in the responses to ensure understanding. 9. Leverage Feedback Mechanisms Provide Feedback: If an explanation seems incorrect or unclear, providing feedback helps in adjusting future responses and improves the learning experience. 10. Encourage the Use of Teaching Aids Request Examples: Ask for real-world applications or examples where the anatomical knowledge is applied, such as in clinical settings, to enhance understanding and relevance. By utilizing these strategies, you can significantly enhance the effectiveness of using AI like me in anatomy education. This approach not only ensures that the information received is accurate and reliable but also supports a deeper engagement with the subject matter.
34	You're welcome! I'm glad to assist in any way that contributes to the growth and understanding of Anatomy. If you have more questions in the future or need further clarification on any topic, feel free to reach out. Happy learning!

## Discussion

Initially, ChatGPT admitted that it can make mistakes and advised us to check. We asked about the roles of ChatGPT in AE. It mentioned various benefits like supplementary learning resources, interactive questions and answers, custom content creation, visualization aid, and 24/7 availability. ChatGPT was acquainted with "Competency Based Undergraduate Curriculum for the Indian Medical Graduate" and its competencies. When we instructed the chatbot to write a short note on “Parts and blood supply of a long bone,” it described very briefly about the parts of bone and omitted the essential details of the arteries of the long bone (Table 2). Similarly, Totlis et al. also observed the lack of anatomical details in responses produced by ChatGPT [11]. Further, when asked to draw its schematic diagram, it generated an incorrect diagram with faulty labeling (Figure 1).

**Figure 7:**
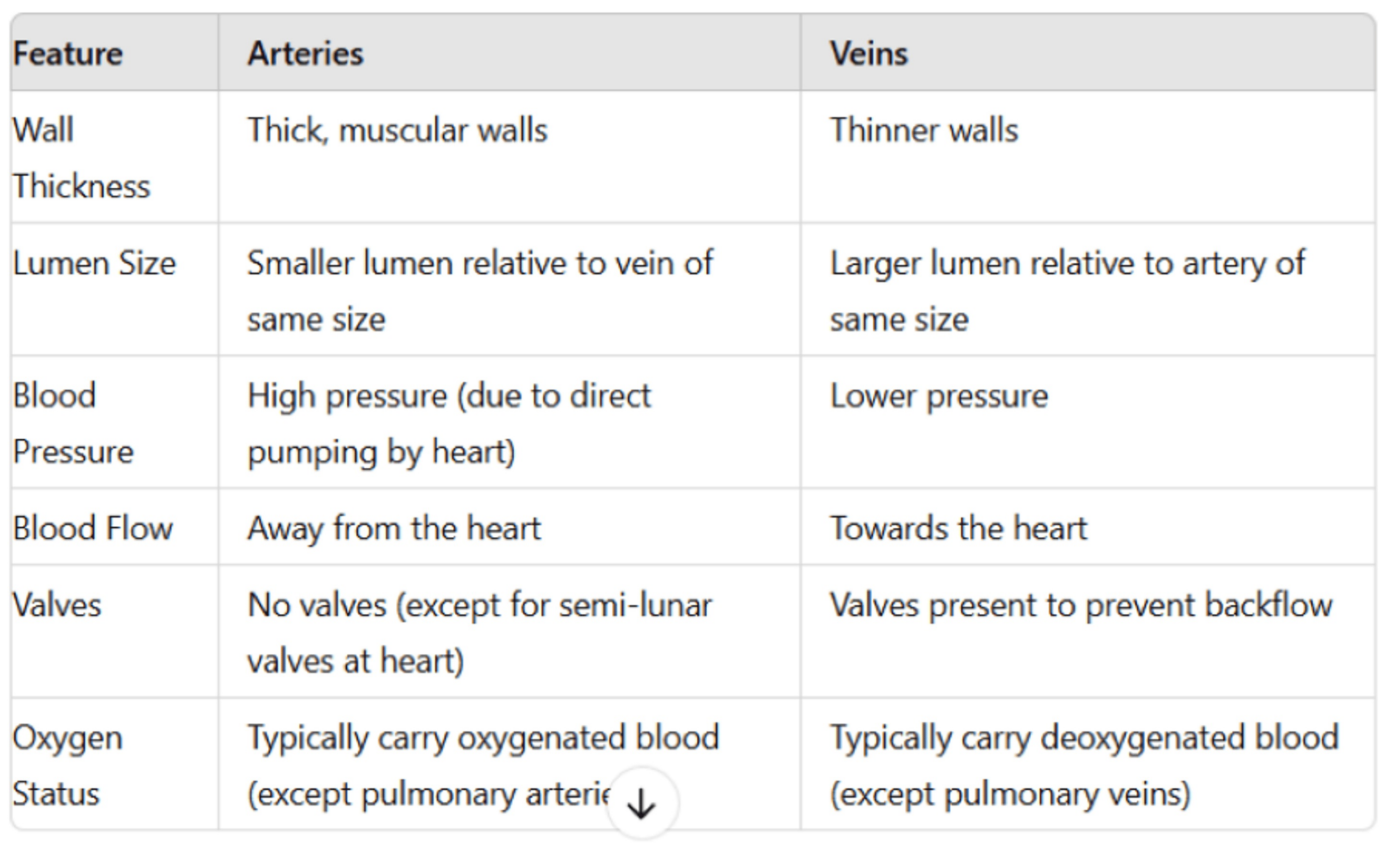
Table generated by ChatGPT.

ChatGPT formed a good table of general differences between arteries and veins (Figure 7). However, it did not comment on the layers of walls of the blood vessels. When asked for an enumeration of peculiarities of the clavicle, the chatbot mentioned its most peculiarities but omitted a few facts like the type of ossification and piercing by supraclavicular nerves. Further, it described the factors of maintaining the arches of the foot in a very concise manner, which is inadequate for MBBS students (Table 2).

Further, we asked ChatGPT to write a model answer on “Describe the anatomical basis of ischemic heart disease.” The chatbot accurately described the anatomical basis, though a brief detail of the arterial supply of the heart should have been mentioned for a five-mark question for MBBS students. Further, the chatbot explained the clinical significance of pterygoid venous plexus precisely but did not point out its detailed communications. However, it described the clinical importance of Calot’s triangle nicely (Table 2).

Considering the importance of transverse sections of the brain stem for MBBS students, we instructed ChatGPT to draw and label transverse sections of pons at the upper and lower levels. It explained these sections in brief, which was inadequate and generated their incorrect illustrations (Figure 2). However, it enumerated parts & major connections of basal ganglia and limbic lobe adequately (Table 2).

When asked to describe the ultrastructure of muscular tissue with a diagram, ChatGPT omitted to mention the shape of skeletal and cardiac muscle fibers, did not touch upon the location of their nucleus, and produced an incorrect diagram (Figure 3). However, the chatbot explained Lyon’s hypothesis very nicely. Similarly, it elucidated the cleavage and formation of blastocyst quite well (Table 2).

**Figure 8:**
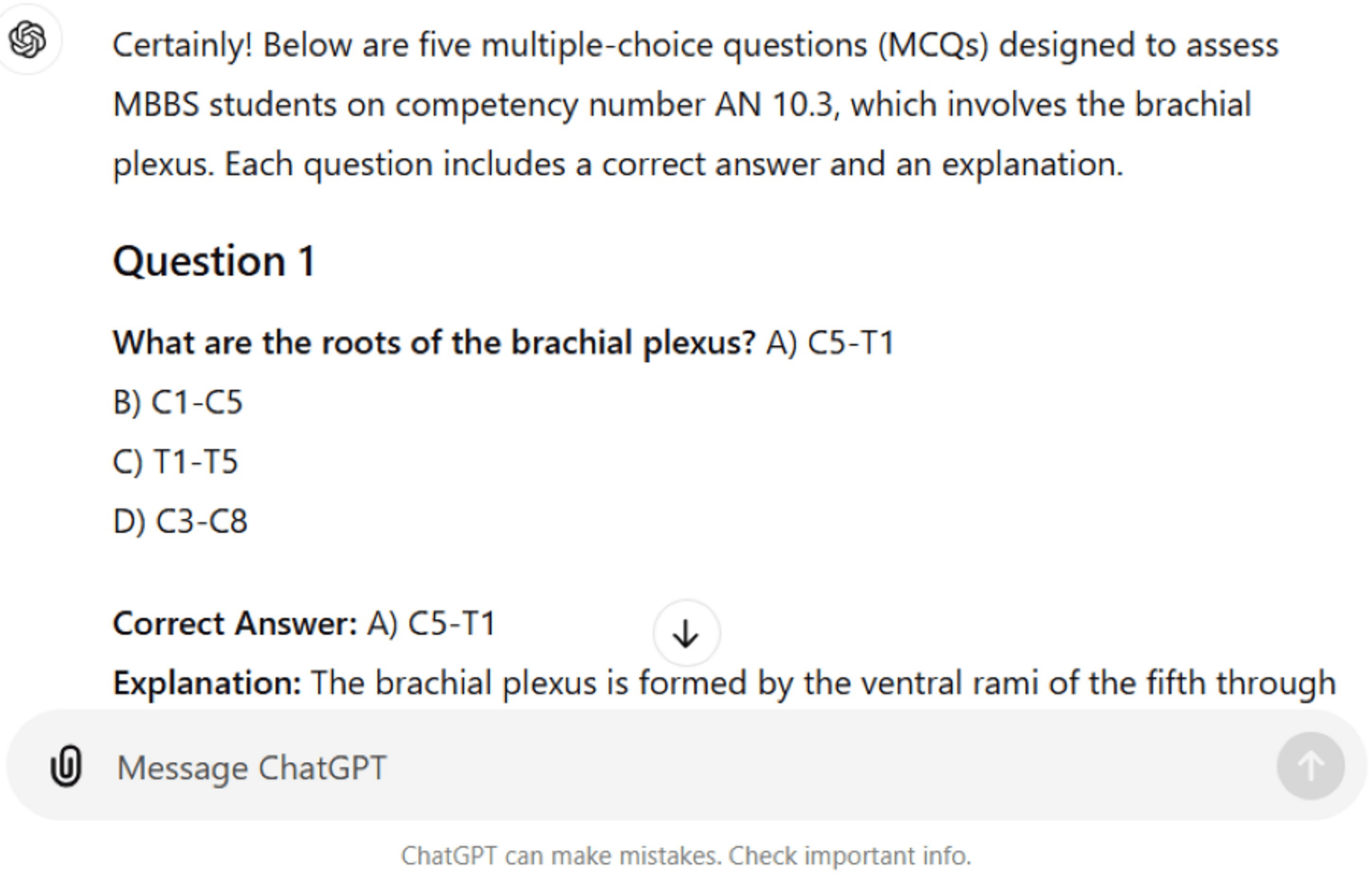
Multiple-choice question created by ChatGPT.

Subsequently, we instructed ChatGPT to make five multiple-choice questions (MCQs) with explanatory answers on the topic of brachial plexus (Figure 8). It was observed that the MCQs were ordinary, though in MCQ No. 3 regarding the artery related to the axillary nerve, a specific artery related to it in quadrangular space could have been inquired. Similarly, in MCQ No. 4 regarding sensory innervations of the thumb, a particular surface of the thumb should have been mentioned to assess MBBS students. Furthermore, the explanations provided with MCQs were also inadequate to rule out incorrect choices (Table 2).

**Figure 9:**
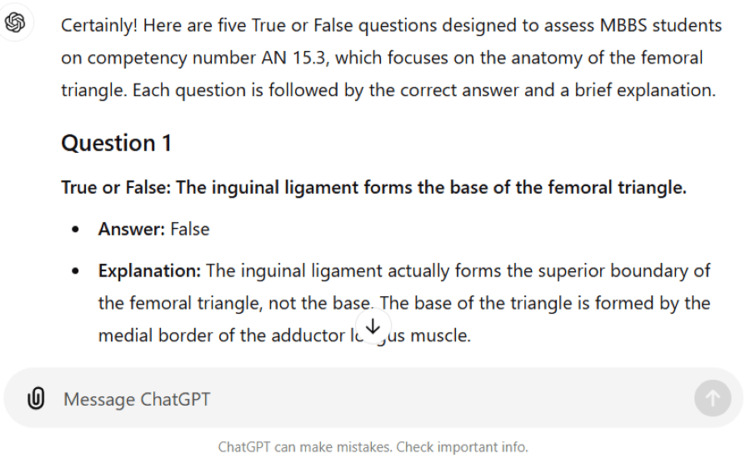
True or False question formulated by ChatGPT.

**Figure 10:**
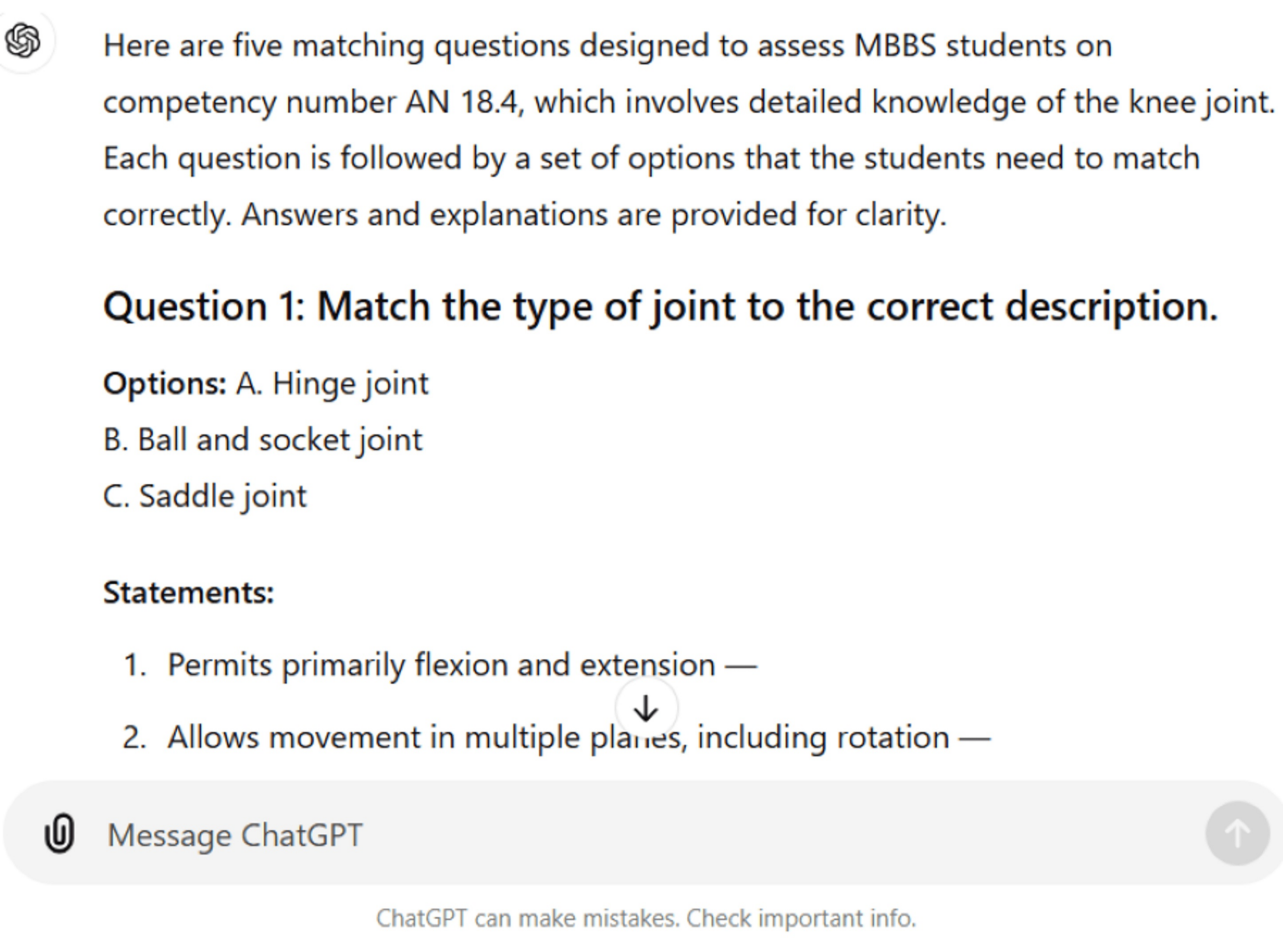
Matching question generated by ChatGPT.

Thereafter, we directed ChatGPT to formulate five true or false questions on the topic of the femoral triangle (Figure 9). The chatbot generated the four correct questions with a good explanation, though the first question regarding the base of the femoral triangle was observed to be incorrect, as the inguinal ligament certainly forms the base of the femoral triangle (Table 2). Further, it generated accurate matching questions regarding knee joints (Figure 10).

**Figure 11:**
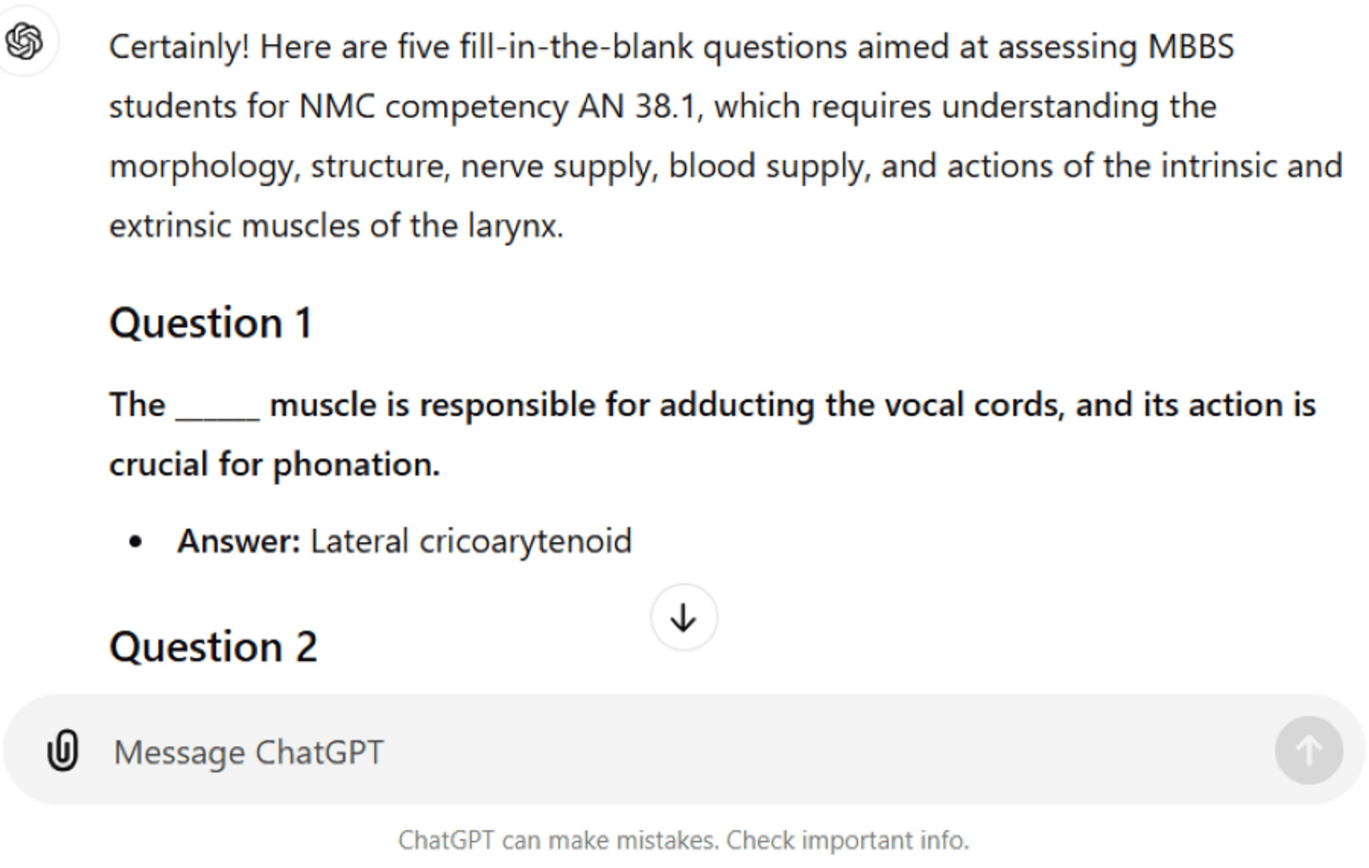
Fill-in-the-blank question generated by ChatGPT.

The chatbot also formulated a good short-answered question on the bronchopulmonary segment, though it did not point out the names of bronchopulmonary segments in each lung. ChatGPT also created a very good case-based MCQ with a detailed explanation of “lateral medullary syndrome.” Thereafter, we asked the chatbot to generate five questions with answers for structured oral viva voce on the ventricles of the brain and it formulated basic questions with precise answers. It also produced good "fill in the blank" questions on the gross anatomy of the larynx (Figure 11 and Table 2).

Then, we instructed ChatGPT to describe the various methods to assess the skill of the medical students on the topic of "blood vessels and nerves of hand" and it beautifully explained a variety of techniques such as cadaveric dissection, objective structured clinical examination (OSCE), case scenario simulation, and peer teaching. Subsequently, we requested the chatbot to provide steps to conduct a DOAP (Demonstration-Observation-Assistance-Performance) session on the temporomandibular joint, it elucidated the first two steps satisfactorily but instead of assistance and performance, it delineated assessment and participation. However, ChatGPT nicely marked out the details of steps to conduct an introductory session of two hours for AETCOM (attitude, ethics, and communication) Module 1.5, focusing on demonstrating respect and following the correct procedures when handling cadavers and other biological tissues (Table 2).

**Figure 12:**
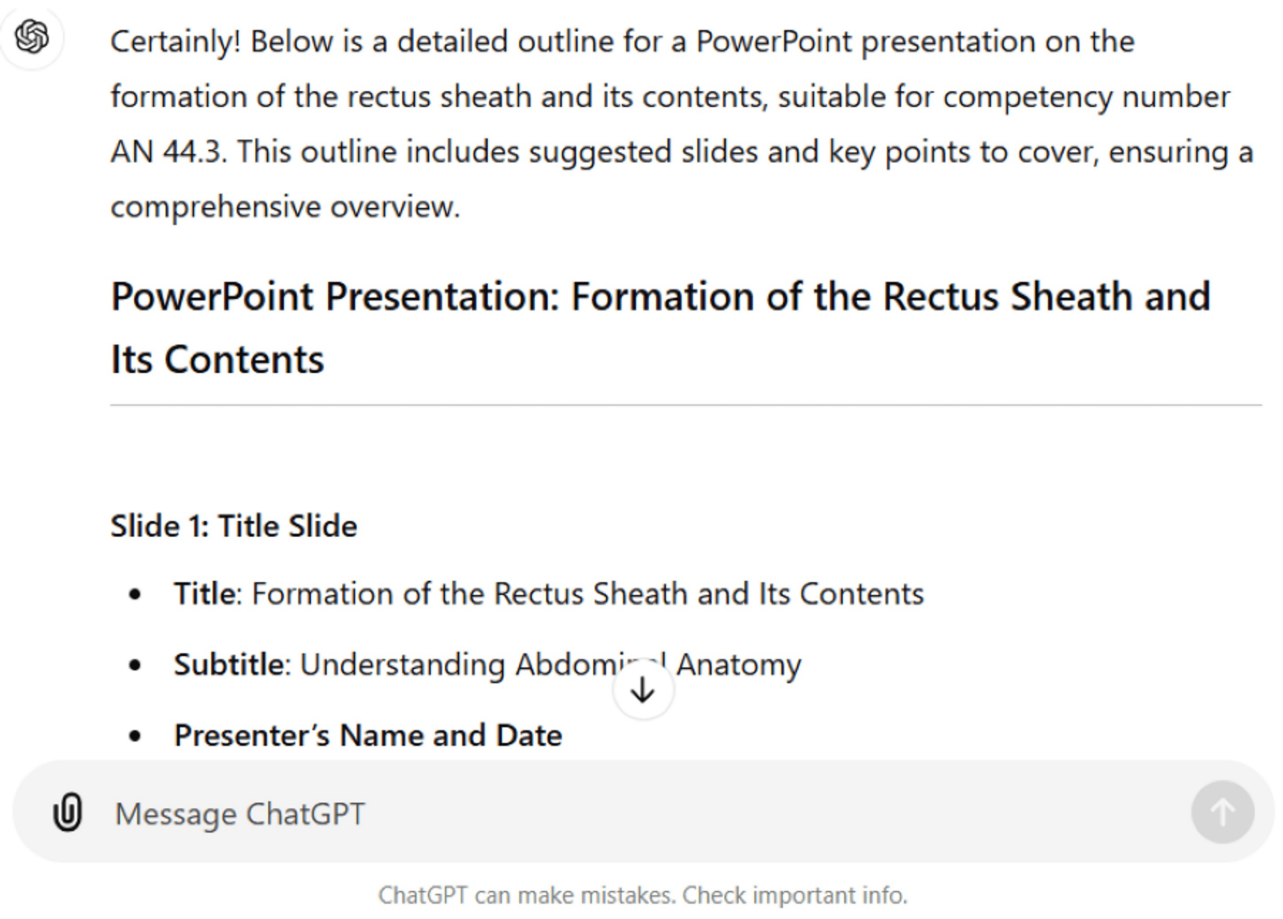
Lecture made by ChatGPT.

Further, we directed ChatGPT to make a lecture for a PowerPoint presentation on the topic of the “Rectus sheath” and it provided a detailed outline of the lecture but could not formulate a good lecture (Figure 12). Then, we instructed the chatbot to evaluate the hypothetical answer written by a medical student regarding the arterial supply of the uterus. It properly evaluated the answer and gave two marks out of five with justification. The chatbot also yielded the recommendations for improvement of student’s answers (Table 2).

Additionally, the chatbot was asked to identify the hand-drawn schematic diagram of the transverse section of the spinal cord, but the chatbot mistakenly identified the figure as the brain's ventricular system (Figure 4). However, it recognized the outline of the stomach without error and was able to determine the arrow-marked part of the stomach (Figures 5, 6). Ultimately, we requested ChatGPT to propose some techniques to get accurate, reliable, and valid answers to improve AE and it suggested asking clear and specific questions, utilizing follow-up questions, specification of the level of details, and providing feedback of the responses (Table 2).

## Conclusions

ChatGPT could be a helpful interactive tool for medical students to understand the clinical significance and details of the anatomical structures if approached in a systematic manner. Though ChatGPT-4 can generate images for various anatomical structures, they do not depict the required details perfectly. Further, the chatbot could be a valuable anatomy tool for medical teachers also, as it creates a variety of quizzes, provides necessary steps to conduct various teaching-learning sessions, generates appropriate outlines of the lectures, and can evaluate the answers written by the students with proper justification. Ultimately, it can be concluded that ChatGPT provides great assistance to anatomy teachers in implementing the curriculum, and complements the role of educators but is not able to replace them.

The current study investigated only a few anatomical topics to evaluate ChatGPT’s probable influence on AE. Consequently, the precision of the chatbot’s response may differ if researchers inquire about various other anatomical structures. We propose further studies to offer recommendations for the best possible utilization of ChatGPT in AE.
